# A Rational Designed PslG With Normal Biofilm Hydrolysis and Enhanced Resistance to Trypsin-Like Protease Digestion

**DOI:** 10.3389/fmicb.2020.00760

**Published:** 2020-05-13

**Authors:** Tiantian Su, Jing He, Ningna Li, Shiheng Liu, Sujuan Xu, Lichuan Gu

**Affiliations:** State Key Laboratory of Microbial Technology, Shandong University, Qingdao, China

**Keywords:** *Pseudomonas aeruginosa*, biofilm, PslG, trypsin, protease

## Abstract

A glycosyl hydrolase produced by *Pseudomonas aeruginosa*, PslG, has become a promising candidate for biofilm treatment because of its ability to inhibit and disperse biofilms by disrupting exopolysaccharide matrix at nanomolar concentrations. However, as a protein, PslG used for treatment may be degraded by the ubiquitous proteases (of which trypsin-like serine proteases are a major group) secreted by human cells. This would lead to an insufficient effective concentration of PslG. Here, based on the result of liquid chromatography–tandem mass spectrometry (LC-MS/MS) and structural analysis, we generate a PslG mutant (K286A/K433S) with greatly enhanced trypsin resistance. This measure raises IC_50_ (the concentration of trypsin that can degrade 50% of protein in 30 min at 37°C) from 0.028 mg mL^–1^ of the wild-type PslG to 0.283 mg mL^–1^ of PslG^*K*286*A/K*433*S*^. In addition, biofilm inhibition assay shows that PslG^*K*286*A*/*K*433*S*^ is much more efficient than wild-type PslG in the presence of trypsin. This indicates that PslG^*K*286*A/K*433*S*^ is a better biofilm inhibitor than wild-type PslG in clinical use where trypsin-like proteases widely exist.

## Introduction

Biofilms are highly structured matrix-enclosed bacterial communities adherent to surfaces (Costerton et al., 1995; Stoodley et al., 2002). Biofilm bacteria have extremely enhanced resistance to antibiotic treatments and host immune responses compared with their planktonic living states (Stewart and Costerton, 2001; Stewart and Franklin, 2008).

*Pseudomonas aeruginosa* is the most common opportunistic pathogen that causes 10–20% of infections in most hospitals and is associated with a high mortality rate up to 61% (Bodey et al., 1983; Kang et al., 2003). *P. aeruginosa* can cause both acute and chronic infections, which could be life-threatening. Chronic infection is more difficult to eradicate because of the formation of biofilms and elevated antibiotic resistance. *P. aeruginosa* has the ability to form biofilms on various surfaces, including biotic and abiotic surfaces (O’Toole et al., 2000). For example, *P. aeruginosa* can colonize on many medical implants such as catheters, ventilator tubes, and contact lenses, which is the major cause of hospital infections (Ahiwale et al., 2011). *P. aeruginosa* is also the leading cause of chronic respiratory infection and lung infections in patients with cystic fibrosis (Waters and Goldberg, 2019). Furthermore, amounting to 60–70% contact lens-related keratitis is caused by *P. aeruginosa*, which causes global blindness and visual impairment (Choy et al., 2008).

To date, the treatment of biofilm infection in clinic is either through mechanical debridement or use of antibiotics, which is arduous and inefficient because biofilm bacteria exhibit extremely high drug tolerance. Because of the critical role of extracellular matrix in biofilm formation and development, enzymes targeting the matrix molecules (proteins, extracellular DNA, and exopolysaccharides) have been gaining more and more attention for novel therapeutics (Mah and OToole, 2001; Kiedrowski et al., 2011; Newell et al., 2011; Moormeier et al., 2014; Fleming et al., 2017; Zhao et al., 2018). DNase I and its derivative DNase1L2 have been reported to prevent biofilm formation of *P. aeruginosa* and *Staphylococcus aureus* at early stage (Whitchurch, 2002; Eckhart et al., 2007). A purified β-*N*-acetylglucosaminidase, dispersin B, is able to disperse biofilms formed by *Aggregatibacter actinomycetemcomitans*, *Staphylococcus epidermidis*, and other species when supplied alone or in synergy with cefamandole nafate (Donelli et al., 2007; Darouiche et al., 2009). Other biofilm-degrading enzymes, including α-amylase, lysostaphin, and alginate lyase, also show antibiofilm activities against various pathogenic bacteria (Alkawash et al., 2006; Kokai-Kun et al., 2009; Kalpana et al., 2012; Singh et al., 2015; Algburi et al., 2017).

Our previous work revealed that a self-produced glycoside hydrolase PslG efficiently prevents biofilm formation and disassembles existing biofilms of a wide range of *Pseudomonas* strains by mainly disrupting the Psl matrix, which makes PslG an important candidate for biofilm treatment (Baker et al., 2015; Yu et al., 2015). Subsequently, Zhang et al. (2018) reported that PslG can affect the surface movement of *P. aeruginosa*. After adding PslG to the medium, bacteria move significantly faster and in a more random way with no clear preferred direction. Besides PslG, other glycoside hydrolases such as PelA have also been reported to exhibit biofilm-disrupting ability. However, compared to PelA, PslG has more advantages. Experimental data showed that PslG at a much lower half maximal effective concentration was more effective than PelA (Baker et al., 2016). Also, PslG does not inhibit bacteria growth and is non-toxic to human epithelial cells and immune cells. Furthermore, PslG treatment sensitized biofilm bacteria to antibiotics and thus can be used together with antibiotics (Yu et al., 2015). Finally, to our experience, the production of PslG is very easy because when expressed in *Escherichia coli* the yield is high.

As the most potent factor for biofilm disassembly and inhibition by far, PslG is gaining increasing attention. Recently, a clinic study using murine and porcine wound models showed that PslG can be used to treat *P. aeruginosa*-infected wounds by improving antibiotic efficacy and host innate immune activity (Pestrak et al., 2019). Taken together, these results showed that PslG has promising potential to be used in clinic to combat biofilm-related infections, such as in burned patients, bacterial keratitis, and respiratory system and gastrointestinal tract infections. However, PslG has intrinsic disadvantages as a protein, because it could be digested by the ubiquitous proteases secreted by human cells. This may severely reduce the lifetime of PslG, thus restricting the enzyme activity. It has been reported that ∼2% of the genes in human genome encode proteases, of which trypsin-like serine proteases are a major group (Overall and Blobel, 2007; Wang et al., 2018). In addition, *P. aeruginosa* itself secretes several extracellular proteases such as protease IV and Ps-1, which are important virulence factors and have been characterized as trypsin-like proteases (Elliott and Cohen, 1986; Engel et al., 1998). Thus, development of trypsin-resistant variant may significantly improve PslG activity in clinical usage. In this study, we generate a liquid chromatography–tandem mass spectrometry (LC-MS/MS) and three-dimensional (3D) structure-based rational design of PslG to enhance its resistance to trypsin. By introducing double mutations K286A and K433S, the resistance of PslG to trypsin was significantly increased, and so was the biofilm inhibition activity in the presence of trypsin.

## Materials and Methods

### Strains and Growth Conditions

*Pseudomonas aeruginosa* PAO1 was grown at 37°C in Luria–Bertani (LB) broth (Becton Dickinson, Franklin Lakes, NJ, United States) without sodium chloride (LBNS). Biofilms of *P. aeruginosa* were grown at 30°C in Jensen’s medium, a chemically defined medium (Jensen et al., 1980). *E. coli* BL21 (DE3) was grown in LB broth at 37°C. All the safety procedures of institution biosafety level 2 standard were adhered to while working with *P. aeruginosa* PAO1.

### Plasmid Construction and Protein Purification

Sequence encoding PslG (residues 31–442) was amplified from *P. aeruginosa* PAO1 genome DNA and cloned into PGL01, a vector modified from pET15b with a PreScission Protease (PPase) cleavage site for the removal of the His-tag. Plasmids expressing PslG with single or double mutations were constructed by using QuickChange with wild-type PGL01-*PslG* as the template (Xia et al., 2015). The primers used for QuickChange are listed in Supplementary Table S1.

*Escherichia coli* BL21 (DE3) cells expressing wild-type or mutant PslG were cultured in LB medium supplemented with 100 μg mL^–1^ ampicillin at 37°C until OD_600_ reached 0.8 and were induced overnight with 0.12 mM isopropyl β-D-thiogalactopyranoside at 22°C. For protein purification, bacterial cells were harvested and resuspended in binding buffer (25 mM Tris pH 8.0, 200 mM NaCl) and lyzed by sonication. After centrifugation at 4°C for 45 min, the supernatants were loaded onto a nickel affinity column (Chelating Sepharose Fast Flow; GE Healthcare, Chicago, IL, United States) and washed with binding buffer to remove the non-specific bindings. Then, the resins were resuspended in binding buffer and incubated with 0.12 mg mL^–1^ PPase overnight at 4°C to remove the His tag. After elution with binding buffer, the protein samples were further purified by ion-exchange chromatography (Source 15Q HR 16/10; GE Healthcare) using gradient elution with 0-1 M NaCl, 25 mM Tris pH 8.0. Finally, the protein sample was purified by size-exclusion chromatography (Superdex 200 10/300 GL; GE Healthcare) in buffer containing 100 mM NaCl, 10 mM Tris pH 8.0.

### Protein Digestion and LC-MS/MS Analysis

The sodium dodecyl sulfate (SDS) gel band of PslG was collected, washed with water, and destained in destaining buffer containing 50% acetonitrile (ACN) and 25 mM ammonium bicarbonate. The disulfide bonds from cysteinyl residues were reduced with 10 mM DTT for 1 h at 56°C, and then the thiol groups were alkylated with 55 mM iodoacetamide for 45 min at room temperature in darkness. The gel band was washed in turn with 25 mM ammonium bicarbonate and destaining buffer. All the liquids were removed, and the gel piece was dried in a SpeedVac, Eppendorf, Hamburg, Gemany. The dried gel band was reswollen and covered with 25 mM ammonium bicarbonate containing 67 ng μL^–1^ trypsin and was incubated overnight at 37°C. The digestion was stopped by the addition of 0.1% formic acid (FA). Ten microliters of the resulting sample was loaded onto a C18 column using a gradient buffer containing 5–80% ACN and 0.1% FA. The peptides were then subjected to MS/MS analysis using MicrOTOF-QII (Bruker Daltonics, Billerica, MA, United States). The MS data were analyzed and matched to the protein sequence of PslG by Mascot search engine version 2.3.01.

### Trypsin Proteolysis Analysis

Wild-type and mutant PslG proteins were either concentrated or diluted to 2.7 mg mL^–1^ with 10 mM Tris-HCl pH 8.0 and 100 mM NaCl. Trypsin from bovine pancreas powder was bought from Sigma–Aldrich, St. Louis, MI, United States and was made into stock solution at the concentration of 80 mg mL^–1^ with buffer containing 10 mM Tris-HCl pH 8.0 and 100 mM NaCl. Working solutions of trypsin were obtained through serial dilution using the same buffer. Equal volumes of PslG and trypsin solutions were mixed and incubated at 37°C for 30 min. 2 × SDS loading buffer was added to the mixture to stop the reaction. The resulting samples were then loaded onto SDS–polyacrylamide gel electrophoresis (PAGE), stained with Coomassie brilliant blue, and quantified by densitometric scanning using ImageJ software^1^. The experiments were repeated for at least three times with similar results, and representative images are shown.

### Biofilm Inhibition and Disassembly Assay

For biofilm inhibition assay, an overnight culture of PAO1 was diluted 100 times and inoculated into Jensen’s medium containing 50 nM wild-type or mutant PslG in a microtiter dish (Flacon 3911). After 24 h of static growth at 30°C, the medium and planktonic cells were discarded, and the wells were washed three times with water. The biofilms were stained with 0.1% crystal violet (CV) and washed three times with water. Then, the CV bound to biofilms was solubilized with 30% acetic acid and measured for absorbance at 560 nm. For disassembly assay, the culture was replaced by fresh medium containing 50 nM PslG after 24-h growth in Jensen’s medium without PslG. After 30-min treatment at 30°C, the biofilm abundance was measured in the same way. For trypsin treatment experiments, trypsin was added to PslG at indicated concentrations. The sample without PslG was used as an untreated control. For these experiments, data of three independent repeats are shown in the figures as means ± SD.

### Structural Modeling

The structural modeling of PslG^*K*286*A/K*433*S*^ was constructed using online server ITASSER (Zhang, 2008; Roy et al., 2010; Yang et al., 2015). The final model with the highest C score was selected from the top five models predicted. Structural alignment and figures were made using PyMOL.

## Results

### LC-MS/MS and Structure-Based Selection of Proper Trypsin Cleavage Sites

PslG is predicted to be a periplasmic protein, resembling β-D-xylosidases from the CAZy glycosyl hydrolase family 39 (Friedman and Kolter, 2004; Franklin et al., 2011). The full-length protein of PslG contains 442 amino acids. The first 30 amino acids are predicted to be the signal peptide by SignalP 5.0. In our previous work, the crystal structure of PslG (amino acids 31–442) was solved at 2.0 Å, which consists of an N-terminal catalytic domain and a C-terminal carbohydrate-binding domain. Glu165 and Glu276 are the key catalytic residues (Yu et al., 2015). In this study, through combination of structural analysis and LC-MS/MS, we are able to design a trypsin-resistant mutant of PslG.

**FIGURE 1:**
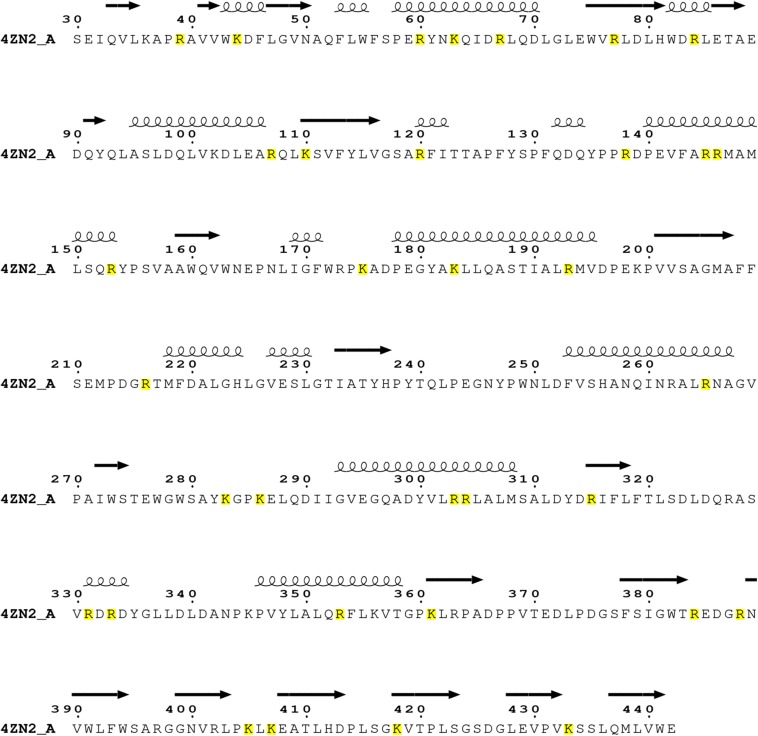
Sequence analysis on the trypsin cleavage residues (highlighted with yellow color) in PslG (PDB: 4ZN2) identified through LC-MS/MS. The catalytic domain of PslG includes residues 47–358, whereas the carbohydrate-binding domain (CBM) of PslG consists of residues 31–42 and 361–442.

**FIGURE 2:**
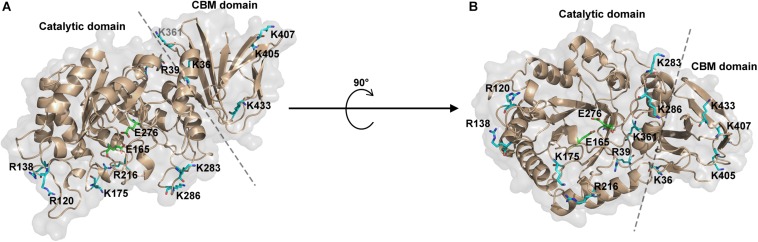
Representation of potential trypsin cleavage sites. **(A,B)** The structure of PslG is shown in cartoon and surface modes. The catalytic domain and carbohydrate-binding domain (CBM) are separated by a dashed line. Surface residues related to trypsin proteolysis are shown in sticks and colored as blue. The key catalytic residues (E165 and E276) of PslG are shown in stick and colored as green.

As known, trypsin cleaves peptides on the C-terminal side of lysine or arginine, except that there is a proline residue on the carboxyl side of the cleavage site. For initial screening of potential trypsin cleavage sites, PslG protein was digested with trypsin and subjected to LC-MS/MS analysis. Mascot search engine was used to match detected peptides to PslG amino acid sequences. Through this method, we found 34 potential cleavage sites in PslG, which are listed in Figure 1. According to our structure, 11 (R39, R120, R138, K175, R216, K283, K286, K361, K405, K407, and K433) of the 34 residues reside in the loop region other than in α-helices and β-strands, which contribute more to protein folding (Figure 2). Because PslG is a monomer in solution (Baker et al., 2015; Yu et al., 2015), residues located on the surface are exposed to solvent, thus making them more favorable for trypsin digestion. Together, we chose the residues that are most external, flexible, and far away from the catalytic site for mutation analysis. These mutants include single mutations R39Q, R120S, K175S, K283A, K361S, K405N, K433S, and one double mutation K286A/K433S. We also include K36A fully based on the structural analysis. Among these residues, K361, K405, and K433 are located in the C terminal carbohydrate-binding domain of PslG, and the others are located in the catalytic domain.

### Trypsin–Proteolysis Analysis of Wild-Type and Mutant PslG

**FIGURE 3:**
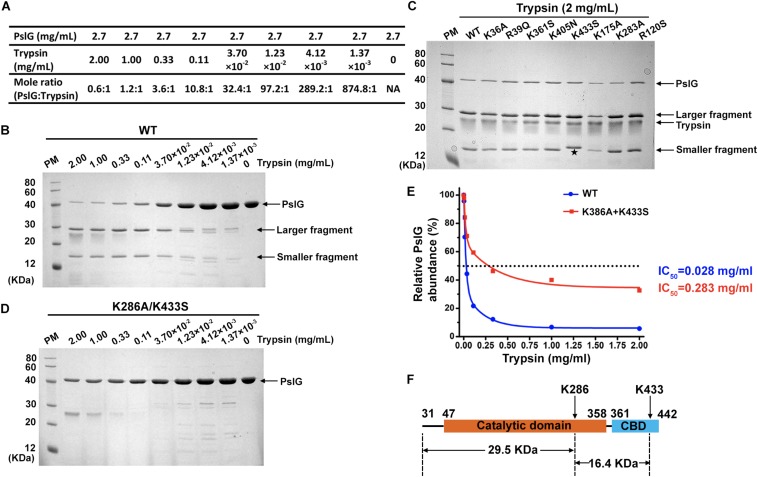
Trypsin proteolysis of wild-type and mutant PslG. **(A)** The corresponding molar ratios of PslG to trypsin used in the experiments are shown. **(B)** The proteolysis of wild-type PslG treated with different concentrations of trypsin for 30 min at 37°C. The larger and smaller stable bands generated after digestion are marked by arrows. **(C)** Sodium dodecyl sulfate–PAGE showing the proteolysis characteristics of wild-type and single mutant PslG variants when treated with 2 mg mL^– 1^ trypsin for 30 min at 37°C. The smaller fragment generated by PslG^*K*433*S*^ is marked with an asterisk. **(D)** The proteolysis of PslG^*K*286*A/K*433*S*^ by different concentrations of trypsin for 30 min at 37°C. **(E)** Quantification of PslG abundances in **(B)** and **(D)** using ImageJ software. The highest PslG intensity was set as 100%. **(F)** The diagram showing trypsin cleavage in PslG.

Recombinant proteins of wild-type and mutant PslG (31–442 residues) were purified from *E. coli* BL21 (DE3). All the mutant proteins showed similar expression level and good solubility comparable to wild-type PslG (Figure 3C). In order to determine the resistance of PslG mutations against trypsin, we evaluated the proteolysis of wild-type and mutant PslG with a serial dilution of trypsin (Figure 3A). After digestion, the protein samples were analyzed by SDS-PAGE. Our results revealed that wild-type PslG was sensitive to trypsin even at a very low trypsin concentration (3.70 × 10^–2^ mg/mL) (Figure 3B). The abundance of wild-type PslG was significantly decreased by approximately 85% when treated with 0.33 mg/mL trypsin. As shown, PslG was gradually hydrolyzed into two stable fragments, the molecular weights of which were approximately 30 and 16 kD, respectively.

The proteolysis characteristics of mutant proteins were then examined under the same condition. Except K433S, all the other single mutants showed no obvious difference compared with wild-type PslG when treated with indicated concentrations of trypsin (Figure 3C and Supplementary Figure S1). Interestingly, molecular weight of the smaller fragment was slightly increased in K433S (Figure 3C), which revealed that this mutation has an effect on the proteolysis of PslG. Because PslG was hydrolyzed into two fragments with molecular weights of 30 and 16 kD, we speculated that the related cleavage site may be located near residue 300. According to our previous LC-MS/MS analysis, this site will be either K283 or K286. Because single-mutation K283A did not affect the proteolysis of PslG (Figure 3C), a double-mutant K286A/K433S was constructed and subjected to trypsin digestion assay. Further evaluation showed that the double-mutant (K286A/K433S) had significantly increased resistance against trypsin (Figure 3D). IC_50_ (the concentration of trypsin that can degrade 50% of protein) of wild-type PslG and PslG^*K*286*A*/*K*433*S*^ were approximately 0.028 and 0.283 mg mL^–1^, respectively (Figure 3E), indicating an increase of an order of magnitude in resistance resulting from the double mutation. The abundance of wild-type PslG was reduced to below 10% when treated with 1 or 2 mg mL^–1^ trypsin. However, the protein abundance of PslG^*K*286*A*/*K*433*S*^ remained approximately 40 and 30% after treatment with 1 and 2 mg mL^–1^ trypsin, respectively. As expected, we found that the molecular weights of fragments 31–286 and 287–433 precisely match those of the stable bands found in the proteolysis of the wild-type PslG. These two bands were not observed in the proteolysis of PslG^*K*286*A/K*433*S*^. In accordance with this, the single-mutant K286A also showed greatly increased resistance to trypsin (Supplementary Figure S2). And the stable bands generated by trypsin digestion were also missing in the proteolysis of K286A. Taken together, these results indicated that residues K286 and K433 are essential trypsin cleavage sites of PslG, which cut PslG into three fragments: 31–286, 287–433, and 434–442 (Figure 3F). And this also explained why the smaller bands detected in K433S showed a slightly increased molecular weight.

### PslG^*K*286*A*/*K*433*S*^ Showed Increased Activity in Biofilm Inhibition When Supplied With Trypsin

**FIGURE 4:**
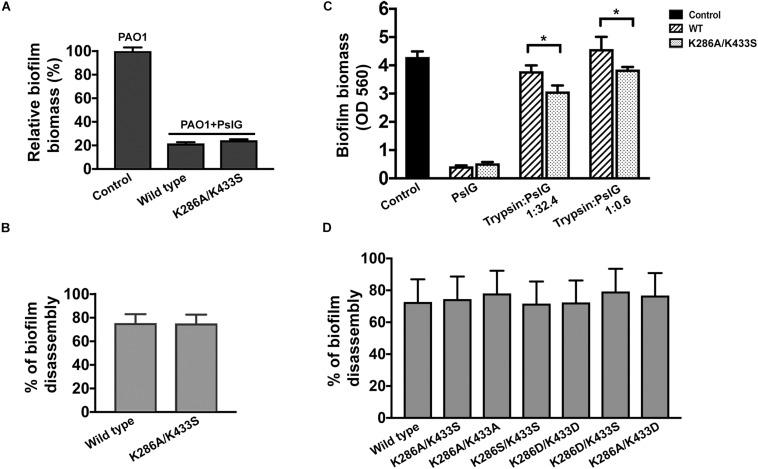
Biofilm inhibition and disperse by wild-type and mutant PslG. **(A)** Biofilm inhibition by wild-type PslG and PslG^*K*286*A/K*433*S*^ in the absence of trypsin. The sample without PslG treatment was used as control and set as 100%. **(B)** Biofilm disassembly by wild-type PslG and PslG^*K*286*A/K*433*S*^. The disassembly rates were calculated using the formula = [(A560 of untreated control - A560 of PslG-treated sample)/A560 of untreated control] × 100%. **(C)** Biofilm inhibition in the presence of different concentrations of trypsin. *T*-test was performed for testing differences between groups. *P < 0.05. **(D)** Biofilm disassembly by PslG K286/K433 mutants. The disassembly rates were calculated as described in **(B)**.

PslG was previously reported to be able to prevent biofilm formation and disassemble existing biofilms while supplied exogenously (Yu et al., 2015). To evaluate the biofilm-inhibiting activity, purified PslG^*K*286*A/K*433*S*^ or wild-type PslG was added into the culture media to a final concentration of 50 nM for inoculation. The biofilm biomass was measured after 24 h of growth. As shown in Figure 4A, the addition of PslG^*K*286*A/K*433*S*^ significantly reduced the biofilm formation of PAO1 by 80%, which was comparable to that of wild-type PslG. The biofilm disassembly activity was examined by incubating preformed biofilms with PslG^*K*286*A/K*433*S*^ or wild-type PslG. After 30-min treatment, both PslG^*K*286*A/K*433*S*^ and wild-type PslG disrupted approximately 75% of biofilms (Figure 4B), which is consistent with our previous results (Yu et al., 2015). Other mutants of these two residues also showed similar biofilm disassembly rate, which indicated that the mutation of K286 and K433 does not affect the enzymatic activity of PslG (Figure 4D).

To determine whether PslG^*K*286*A/K*433*S*^ possesses higher trypsin resistance during biofilm inhibition, two different concentrations of trypsin were inoculated together with PslG (Figure 4C). Although both the wild-type PslG and PslG^*K*286*A/K*433*S*^ showed decreased activities when subjected to trypsin treatment, the remaining activity of PslG^*K*286*A/K*433*S*^ was much higher than the wild-type PslG. When using low concentration of trypsin (trypsin:PslG was 1:32.4), wild-type PslG and PslG^*K*286*A/K*433*S*^ decreased biofilm formation by 10 and 30%, respectively (Figure 4C). When using high concentration of trypsin (trypsin:PslG was 1:0.6), wild-type PslG completely lost biofilm inhibiting activity, whereas PslG^*K*286*A/K*433*S*^ was still able to reduce biofilms by 10% (Figure 4C). These results revealed that PslG^*K*286*A/K*433*S*^ is much more stable and efficient in inhibiting biofilms than wild-type PslG in the presence of trypsin.

## Discussion

PslG was previously reported as a potent factor that can inhibit biofilm formation or disperse existing biofilms of *Pseudomonas* at nanomolar concentrations (Yu et al., 2015). Owing to the potential use of PslG in chronic infection and biofilm treatment, we set out to develop a more stable and active form of this protein. In this study, we found that the double-residue mutant PslG^*K*286*A/K*433*S*^ selected from 35 mutants showed greatly increased resistance to one of the most widespread proteases—trypsin, compared with the wild-type PslG.

**FIGURE 5:**
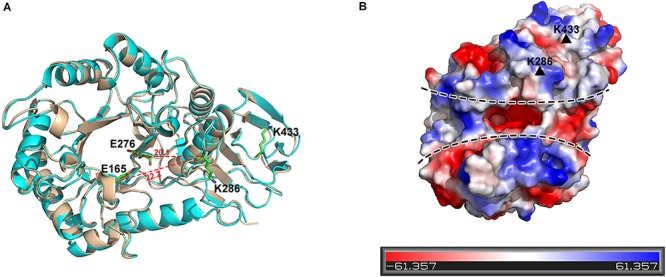
K286 and K433 are spatially far away from the active sites, and their mutants do not affect structure folding. **(A)** Structural alignment of wild-type PslG and PslG^*K*286*A/K*433*S*^. The crystal structure of PslG is shown in wheat. The structure of PslG^*K*286*A/K*433*S*^ was obtained through homologous modeling using ITASSER, and is shown in cyan. The key catalytic residues (E165 and E276) and trypsin hydrolysis sites (K286 and K433) are shown as sticks. **(B)** Electrostatic potential of PslG with negatively and positively charged regions colored in red and blue, respectively. The substrate-binding groove is marked by dashed lines, and residues K286 and K433 are marked by black triangles.

K286 resides in the upper edge of the active cleft of PslG catalytic domain, with its side chain pointing outward, whereas K433 is positioned in the loop area of the carbohydrate-binding domain (Figure 5A). Both of the lysine residues are distal to the active site (e.g., the nearest distance between C-alpha atoms of K286 and the key catalytic residue E276 is 20.1 Å). Additionally, surface potential analysis showed that both K286 and K433 are spatially apart from the negatively charged substrate-binding groove of PslG (Figure 5B). Thus, mutations of these two residues are supposed to have no effect on either catalytic activity or substrate binding of PslG. This is confirmed by the biofilm inhibition and disassembly experiments, indicating that enzymatic activity of PslG^*K*286*A/K*433*S*^ has no difference with the wild-type PslG in the absence of trypsin (Figures 4A,B). While subjected to trypsin treatment, the activity of PslG^*K*286*A/K*433*S*^ is significantly higher than that of wild-type PslG, which revealed that PslG^*K*286*A/K*433*S*^ has higher trypsin resistance (Figure 4C). Single mutants other than K286A and K433S, even though exposed to solvent as well, showed no significant difference compared with wild-type PslG when treated with trypsin. To validate the effect of Ca^2+^ on trypsin activity, we measured the PslG proteolysis with or without Ca^2+^ (Supplementary Figure S3). Our result indicated that calcium ions had no significant effect on the trypsin digestion of PslG. We also constructed a 3D structural model of PslG^*K*286*A/K*433*S*^ through homology modeling using I-TASSER (25–27). This homology model is nearly identical to the structure of wild-type PslG with a *Z* score of 66.7 and root-mean-square deviation (RMSD) of 0.4 (Figure 5A).

## Conclusion

In summary, through a combined experimental and computational approach, we have developed a trypsin-resistant variant of PslG, which is a promising candidate for clinical and environmental biofilm treatment, although the immune response to PslG in clinical usage still needs more research. The enhanced trypsin resistance will lead to increased enzymatic activity of PslG when protease is present, thus promoting lower costs by reducing the dosage of PslG used. It is worth noting that other types of proteases could also affect the biofilm treatment using PslG, such as matrix metallopeptidases, cysteine cathepsins, and pepsin. These proteases may have different cleavage sites compared to trypsin. Future work of PslG mutants with specific resistance to these proteases is desirable by following similar procedure as this article. Our work may also shed light on the structural-based rational design of other enzymes related to biofilm inhibition, such as PelA.

## Data Availability Statement

The datasets generated for this study are available on request to the corresponding author.

## Author Contributions

LG and TS conceived the project. TS, JH, NL, SL, and SX performed the experiments and processed the data. TS analyzed the results and wrote the manuscript. All authors contributed to the editing of the manuscript.

## Conflict of Interest

The authors have filed a patent application on the use of PslG^*K*286*A*/*K*433*S*^. The authors declare that the research was conducted in the absence of any commercial or financial relationships that could be construed as a potential conflict of interest.

## Footnotes

1 http://rsbweb.nih.gov/ij/
