# Supplementary material for: A Rational Designed PslG With Normal Biofilm Hydrolysis and Enhanced Resistance to Trypsin-Like Protease Digestion
Source: Front Microbiol. 2020 May 13;11:760. doi: 10.3389/fmicb.2020.00760 (PMC7237758; doi:10.3389/fmicb.2020.00760)
Supplement: Supplementary file 1 [file Data_Sheet_1.docx]

***Supplementary Material***

**Supplementary Figures and Tables**


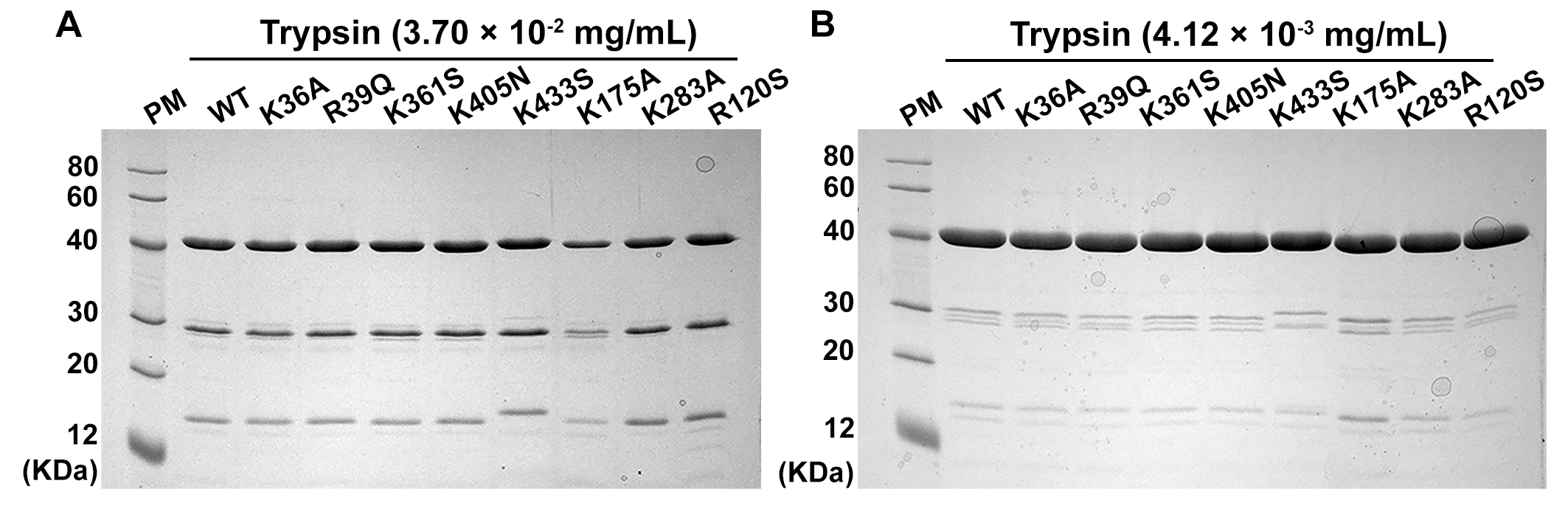


**Supplementary Figure 1.** The proteolysis of wild type and mutant PslG by trypsin for 30 min at 37°C. The concentrations of trypsin used were 3.70×10^-2^ mg mL^-1^ and 4.12×10^-3^ mg mL^-1^, respectively.


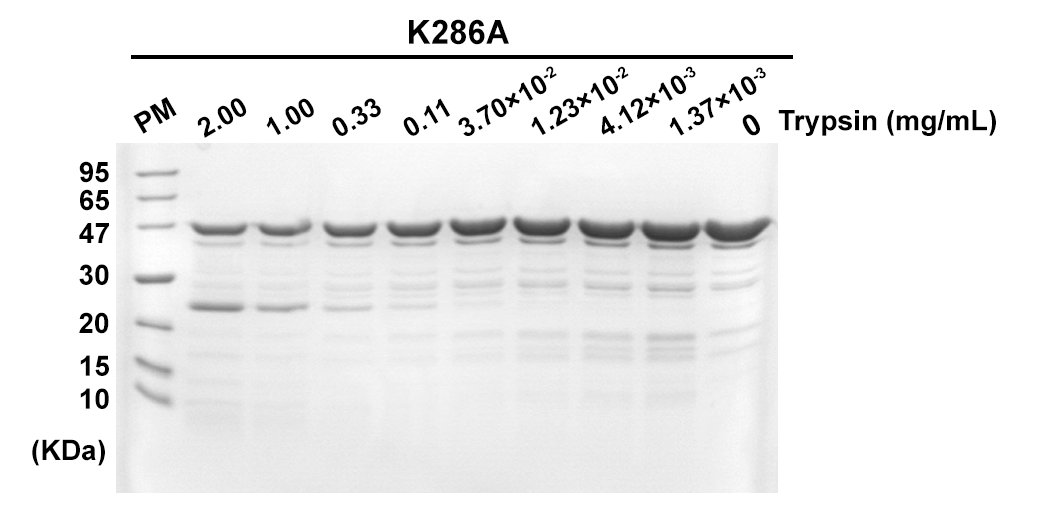


**Supplementary Figure S2.** Trypsin proteolysis of PslG K286A.

**
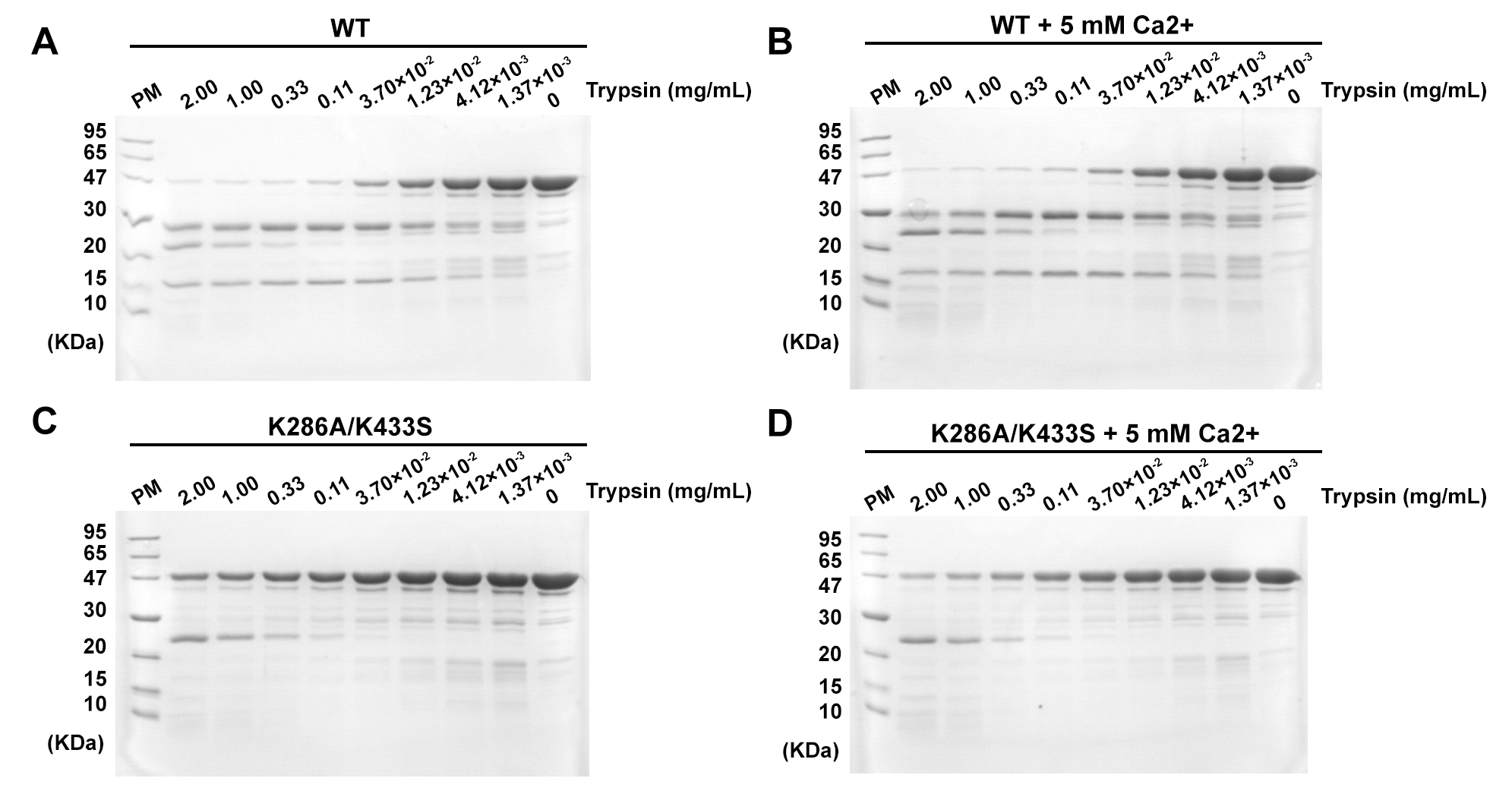
**

Supplementary Figure 3. Trypsin proteolysis of wild-type PslG and double mutant PslG^K286A/K433S^ in the absent and present of Ca^2+^.

**Supplementary Table 1. Primer sequences used to generate the mutants of PslG.**

| **Mutant** | **Forward Primer Sequence (5’-3’)** | **Reverse Primer Sequence (5’-3’)** |
| --- | --- | --- |
| K36A | tactggcagcgcctcgcgcggtggtct | gaggcgctgccagtacctggatctcggcgt |
| R39Q | gcgcctcaagcggtggtctggaaagactt | accaccgcttgaggcgccttcagtacctgga |
| R120S | ctcggccagcttcatcaccaccgcgccgt | tgatgaagctggccgagccgaccaggtaga |
| K175S | ggcccagtgccgacccggaaggctacgccaa | tccgggtcggcactgggccgccagaagccgat |
| K283A | cctacgcagggccgaaggagttgcaggaca | ttcggccctgcgtaggccgaccagccccact |
| K286A | gccggcagagttgcaggacatcattggcgt | atgtcctgcaactctgccggccccttgtag |
| K361S | ggccgagtctgcgcccggccgacccgc | gggcgcagactcggcccggtgaccttgag |
| K405N | tgccgaatctcaaggaggccaccctgcac | tccttgagattcggcaggcgcacgttgc |
| K433S  K286S  K286D  K433A  K433D | cggtgagttccagcctgcagatgctggt  gcctacaaggggccgtcggagttgcagg  gcctacaaggggccggacgagttgcagg  ggcctggaagtcccggtggcctccagcc  ggcctggaagtcccggtggactccagcc | caggctggaactcaccgggacttccagg  gatgtcctgcaactccgacggccccttg  gatgtcctgcaactcgtccggccccttg  cagcatctgcaggctggaggccaccggg  cagcatctgcaggctggagtccaccggg |
